# Experimentally and Modeling Assessment of Parameters Affecting Grinding Aid-Containing Cement–PCE Compatibility: CRA, MARS and AOMA-ANN Methods

**DOI:** 10.3390/polym17111583

**Published:** 2025-06-05

**Authors:** Yahya Kaya, Hasan Tahsin Öztürk, Veysel Kobya, Naz Mardani, Ali Mardani

**Affiliations:** 1 Department of Civil Engineering, Bursa Uludag University, 16059 Bursa, Turkey; 512126007@ogr.uludag.edu.tr (Y.K.); v.kobya@gmail.com (V.K.); 2 Department of Civil Engineering, Faculty of Technology, Karadeniz Technical University, 61080 Trabzon, Turkey; htozturk@ktu.edu.tr; 3 Department of Mathematics Education, Bursa Uludag University, 16059 Bursa, Turkey; nazmardani@uludag.edu.tr

**Keywords:** Marsh funnel flow time, cement–water-reducing admixture compatibility, grinding aids, CRA, MARS, ANN, AOMA-ANN

## Abstract

In this study, the compatibility of polycarboxylate ether-based water-reducing admixtures (PCE) with cements produced with different types and dosages of grinding aids (GA) was experimentally and statistically investigated. A total of 203 paste mixtures were prepared using seven different types of GA and one type of PCE at different dosages. The Marsh funnel flow time and mini-slump values of the mixtures were compared. A modeling study was performed using the experimental data. In this direction, Classical Regression Analysis (CRA), Multivariate Adaptive Regression Splines (MARS), and Artificial Neural Networks (AOMA-ANN) were applied. Innovative approaches, AOMA-ANN (AIP) and AOMA-ANN (ONIP), were introduced. The results showed adverse effects on flow performance with increased GA utilization, except for TEA-based GA. TEA-type GA had the lowest flow performance. AOMA-ANN (ONIP) exhibited the best performance in modeling. Marsh funnel flow-time modeling with AOMA-ANN (ONIP) considered parameters such as sieve residue at 60 microns, the number of molecules per fineness, the density of GA, the pH value of GA, and the PCE dosage. Mini-slump modeling with AOMA-ANN (ONIP) considered parameters such as sieve residue at 60 microns, the density of GA, the pH value of GA, and the PCE dosage.

## 1. Introduction

The cement industry has long been recognized as a major contributor to pollution due to its high consumption of energy and raw materials, and its significant CO_2_ emissions. Recent studies have highlighted the magnitude of these concerns. Notably, it was highlighted that the cement industry accounts for approximately 2% of global electricity consumption [1] and 5% of electricity usage within the industrial sector [2]. Furthermore, cement production is responsible for a substantial portion of global CO_2_ emissions, with estimates ranging from 5% to 7% [3]. To produce one ton of cement, approximately 1.2 tons of raw materials and 130 kWh of energy are expended, resulting in the emission of 1 ton of CO_2_ [4]. Notably, this energy is consumed significantly during the raw material and clinker grinding process [1].

In response to these environmental challenges, various approaches have been explored to mitigate these adverse impacts [5]. Among these approaches, the utilization of grinding aids (GA) during the clinker grinding stage has emerged as a common method to reduce energy consumption [6]. Researchers have also indicated that the use of GA not only enhances grinding efficiency but can also have a profound impact on cementitious systems by altering factors such as the surface properties and particle size distribution of cement particles [7,8]. These alterations in cement properties may, in turn, influence the compatibility of cement with polycarboxylate-based water-reducing admixtures (PCE) [2].

Incompatibility between cement and PCE can result in adverse effects, such as rapid or delayed setting times in cementitious systems [4], segregation [4], loss of consistency, and increased shrinkage [9]. Therefore, it is of paramount importance to assess the compatibility of cement and PCE concerning the fresh state and rheological properties of cementitious systems [10]. One widely employed method for evaluating this compatibility is the determination of Marsh funnel flow time [11]. The Marsh funnel experiment gauges the time it takes for paste mixtures to flow through a standard Marsh funnel [12]. Research has indicated that the Marsh funnel flow time of paste mixtures decreases as the PCE dosage increases, but beyond a certain point, further increases in PCE dosage have negligible effects [13]. This critical point is regarded as the saturation point of PCE [11,14]. Using PCE beyond this saturation point is disadvantageous both economically and in terms of the risk of segregation [11].

While the Marsh funnel experiment is easy and low-cost, the experimental period is long and reproducibility is difficult [15]. To address these issues, researchers have proposed streamlining and enhancing the process through various regression techniques [16]. Regression analysis is a method commonly used to establish relationships between dependent and independent variables, typically assuming linear associations. However, it has been emphasized that many behaviors often involve non-linear relationships [17]. In this regard, Multivariate Adaptive Regression Splines (MARS) offers a non-parametric approach that does not rely on specific assumptions about variable relationships [16,18].

In modeling cement properties, a range of methods have been widely employed, including Artificial Neural Networks (ANN) [19], adaptive neuro-fuzzy inference systems, support vector machines [20], and hybrid approaches utilizing optimization algorithms [21]. ANN has gained popularity and is often integrated with traditional regression or fuzzy logic techniques [22]. More recently, researchers have highlighted the application of various regression techniques, such as MARS [23,24,25,26], M5Tree [24], MARS [26], RF [27], and support vector regression [16]. A summary of recent studies modeling different properties of cementitious systems with hybrid ANNs is presented in Table 1. The studies listed in Table 1 were evaluated focusing on the following four aspects:

(i) *Evaluation in Terms of the Purpose of Using Metaheuristics in ANNs:* When the studies listed in Table 1 are evaluated in terms of the purpose of using metaheuristics with ANNs, metaheuristics were shown to be used in the training of ANNs in the vast majority of studies. In such studies, the optimal values of the weights and biases utilized in the ANN model are identified via metaheuristic algorithms. However, such applications typically result in increased computational times compared to back-propagation algorithms. This is due to the fact that back-propagation algorithms reduce the error obtained in each iteration by back-propagation, whereas the process followed by metaheuristics is a stochastic process. This increases the time required to obtain a lower error. Consequently, in this study, instead of using metaheuristics in training, we used metaheuristic algorithms in the optimization of the network architecture. The design of network architectures is typically user-driven and is often conducted through a trial-and-error process, which can result in the creation of suboptimal network architectures.
(ii) *Evaluation in Terms of the Metaheuristic Algorithms Used:* Upon evaluation of the studies listed in Table 1 in terms of the metaheuristic algorithms employed, it becomes evident that a number of the studies utilize algorithms that are relatively outdated, including Genetic Algorithm (GA), Particle Swarm Optimization Algorithm (PSO), and Differential Evolution (DE). In addition, meta-heuristic algorithms such as Biogeography-Based Optimization (BBO), Covariance Matrix Adapted Evolution Strategy Algorithm (CMAES), Adam Optimizer, Water Cycle Algorithm (WCA), Sine Cosine Algorithm (SCA, 2016), Cuttlefish Optimization Algorithm (CFOA), Electromagnetic Field Optimization (EFO), Runge–Kutta Optimization (RUN), Whale Optimization Algorithm (WOA), Salp Swarm Algorithm (SSA), Grey Wolf Optimizer (GWO), Harris Hawks Optimization (HHO), Slime Mould Algorithm (SMA), Beta Differential Evolution–Improve Particle Swarm Optimization Algorithm (BDE-IPSO), Fruit Fly Optimization Algorithm (FOA), Lion Swarm Optimization Algorithm (LSO), Sparrow Search Algorithm (SSA), Fire Fly Algorithm (FFA), Particle Swarm Optimization Algorithm with Two Differential Mutations (PSOTD) were used in these studies.
(iii) *Evaluation in Terms of Input Parameter Reduction Process:* Upon analysis of the studies listed in Table 1 regarding the aim of reducing ANN input parameters, no study on reducing input parameters could be identified in recent ANN modeling studies on cementitious systems.
(iv) *Evaluation in Terms of the Proposed Metaheuristic Algorithm:* When the studies listed in Table 1 are evaluated in terms of the proposed metaheuristic algorithm, it is evident that a comparison between the metaheuristic algorithms utilized cannot be made, as a significant number of studies employ only a single algorithm. It is observed that the GA, EFO, RUN, LSO, and PSOTD algorithms are proposed for hybrid ANNs in the studies where a comparison is made.

**Table 1:** Recent studies using hybrid ANN methods in cementitious systems.

Reference	Year	Purpose of Using Metaheuristics in ANNs	List of the Metaheuristic Algorithms Employed, Along with the Year in Which They Were First Utilized.	Is There an Input Parameter Reduction Process?	Proposed Metaheuristic Algorithm (If More Than One Algorithm Is Used)
[28]	2024	Training of ANN	BBO (2008)	N/A	N/A
[29]	2024	Training of ANN	PSO (1995)	N/A	N/A
[30]	2023	ANN hyperparameter optimization	CMAES (2001), GA (1989), PSO (1995)	N/A	GA
[31]	2023	Training of ANN	PSO (1995)	N/A	N/A
[32]	2023	DNN hyperparameter optimization	Adam Optimizer (2015)	N/A	N/A
[33]	2023	Training of ANN	WCA (2012), SCA (2016), CFOA (2019), EFO (2016)	N/A	EFO
[34]	2023	Training of ANN	RUN (2021), WOA (2016), SSA (2017), GWO, HHO (2019), SMA (2020)	N/A	RUN
[35]	2023	Training of ANN	BDE-IPSO (2022)	N/A	N/A
[36]	2023	Training of ANN	PSO (1995), FOA (2012), LSO (2018) and SSA (2020)	N/A	LSO
[37]	2022	Training of ANN	GA (1989)	N/A	N/A
[38]	2022	Training of ANN	FFA (2009)	N/A	N/A
[39]	2022	ANN hyperparameter optimization	PSO (1995)	N/A	N/A
[40]	2022	Training of ANN	DE (1997), PSO (1995), PSOTD (2017)	N/A	PSOTD

As a result of these assessments, the gaps identified in the literature are discussed under the following two items:

(i) *The input parameter reduction process has not been previously employed in cement systems:* The input parameter reduction process utilized in this study is an optimization process in which input parameters of low importance are removed from the model, resulting in models with reduced complexity and an enhanced fit to the data.
(ii) *The metaheuristic algorithms used in this study have not been used in previous recent studies:* Gaining–Sharing Knowledge (GSK)-based, JAYA, Symbiotic Organisms Search (SOS), Teaching–Learning-Based Artificial Bee Colony (TLABC), and Teaching–Learning-Based Optimization Algorithm (TLBO) algorithms, which will be used in the optimization of ANN architecture in this study, have not been used and their performance has not been tested.

An extensive literature review indicates that diverse methodologies have been employed to model various aspects of cementitious systems. Nevertheless, it is worth noting that, owing to the intricate nature of these systems and the multitude of influential parameters involved, a definitive consensus remains elusive in this area, as highlighted in previous studies [16,41]. Additionally, research dedicated to modeling the flow behavior of cementitious systems is relatively limited in quantity. Notably, there is a notable absence of studies focused on modeling systems containing GA. This study aimed to investigate the compatibility of cement and PCE in the presence of GAs, which change the properties of cement. In this direction, regression methods were used as an alternative to the Marsh funnel flow experiment, which has a long experimental period. To investigate the effect of PCE–cement compatibility, the Marsh funnel flow time and mini-slump values of paste mixtures were determined using a combination of experimental and statistical approaches. For this purpose, a total of 29 cements containing different types and dosages of GA and 203 different paste mixtures were prepared. As part of the study, Marsh funnel flow time and mini-slump values were independently modeled using regression and hybrid artificial intelligence methods. In the modeling phase, initial equations representing the experimental data were derived through Classical Regression Analysis (CRA) and the Multivariate Adaptive Regression Splines (MARS) method. Subsequently, the error values of the experimental data obtained from these regression models were compared with those acquired through the Artificial Neural Networks with the Architectures Optimized by Metaheuristic Algorithms (AOMA-ANN) method. The AOMA-ANN method was introduced for the first time in this study’s modeling to create numerical models that more accurately reflect the experimental results, achieving lower error rates compared to the CRA and MARS methods. During the modeling phase, initial equations representing the experimental data were formulated using Classical Regression Analysis (CRA) and the Multivariate Adaptive Regression Splines (MARS) method. Then, the error values derived from these regression models were compared with those obtained using the Artificial Neural Networks Optimized by Metaheuristic Algorithms (AOMA-ANN) method. The aim was to address a gap in the field by applying a method that had not been previously documented in the literature.

## 2. Materials and Methods

### 2.1. Materials

In the context of this study, cements containing diverse types and ratios of GA were produced. To achieve this, control cement was obtained through the grinding of a 96% clinker blended with 4% gypsum in a laboratory-type ball mill. In addition to the control cement, seven distinct types of cement were prepared, incorporating different types of GA during the grinding processes. These GAs comprised commonly used commercial variants such as triethanolamine (TEA), triisopropanolamine (TIPA), diethanolisopropanolamine (DEIPA), diethyleneglycol (DEG), and ethyleneglycol (EG). Additionally, two distinct modified TEAs, resulting from the esterification reaction of TEA, were introduced into the mix. In total, seven diverse GAs were incorporated into the cement mixture during the cement grinding stage. All GAs were utilized at varying proportions of 0.025%, 0.05%, 0.075%, and 0.1% relative to the total mass of clinker and gypsum. Consequently, apart from the control cement containing no GA, a total of 29 unique CEM I 42.5R-type cements were manufactured per the EN 197-1 Standard [42]. All cements were ground until they reached the target Blaine fineness value of 4100 ± 100 cm^2^/g. Detailed information regarding the Blaine fineness value, particle size distribution, and zeta potential value of the produced cements is provided in Table 2.

To attain the desired flow performance in this study, a single type of polycarboxylate-based water-reducing admixture (PCE) was employed. Table 3 presents the relevant characteristics of the GAs and PCE used, as supplied by the manufacturer.

### 2.2. Method

In this study, the influence of grinding aid (GA) on cement–PCE compatibility was investigated using Marsh funnel and mini-slump tests (UTEST, Türkiye, 2023). The experiments in question were conducted using the methods suggested by Aïtcin [11] and Kantro [43], respectively. For this purpose, paste mixtures with a water/cement ratio of 0.35 were prepared. In selecting this w/c ratio, existing studies in the literature were taken into account [4,44,45]. The synthesis reaction involves using a carboxylic acid and alcohol, in the presence of heat and a catalyst, to form an ester.Here, the esterification aimed to replace the hydroxyl groups of TEA with a more polar carboxyl group. Two mono-carboxylic acids with short (1–3 carbons) and medium (4–8 carbons) chain lengths were used to modify TEA. A suitable catalyst was identified through preliminary experiments based on the reaction characteristics of TEA and the selected acids.

## 3. Methodology of Modeling

The results obtained from the experimental studies were used in the modeling study. Here, the methods applied in the modeling study are explained.

### 3.1. Development of Models

**Figure 1:**
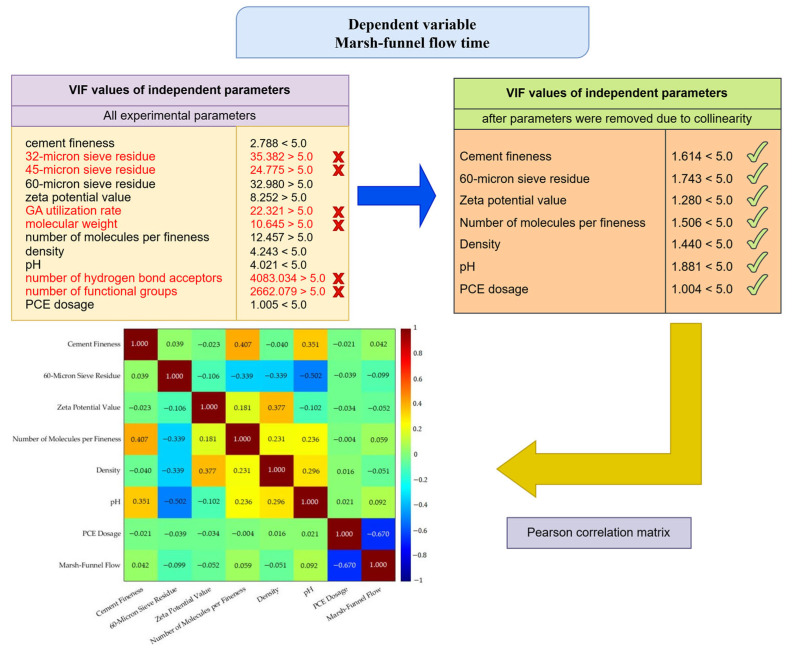
VIF analysis findings and Pearson correlation matrix of experimental data for the Marsh funnel flow-time model.

**Figure 2:**
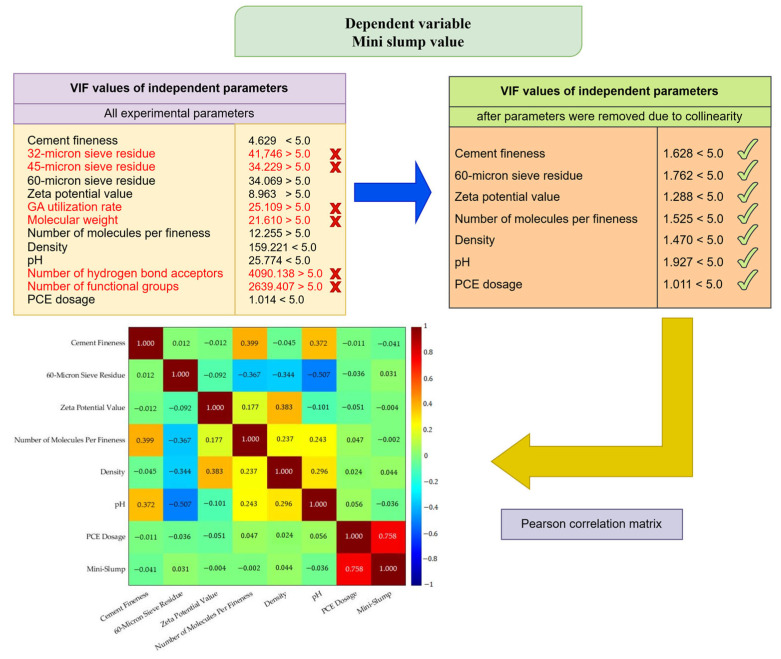
VIF analysis findings and Pearson correlation matrix of experimental data for mini slump value model.

Various modeling techniques were employed in this study, including Classical Regression Analysis (CRA), Multivariate Adaptive Regression Splines (MARS), and Artificial Neural Networks with Architecture Optimized using Metaheuristic Algorithms (AOMA-ANN). As Classical Regression is regarded as a foundational approach to modeling, it is pertinent to illustrate the extent to which the AOMA-ANN method proposed in this study enhances the classical regression method. The second modeling method used in the study, the MARS method, was chosen because it is a powerful modeling method and can be easily applied to the data [46]. The third method used in the study is the ANN method. The superiority of ANNs compared to other machine learning methods, such as Random Forest (RF), Decision Tree (DT), K-Nearest Neighbour (KNN), and Support Vector Machines (SVM), has been proven in many studies [12,47,48,49,50]. The AOMA-ANN method developed in this study enables the automatic and efficient determination of network hyperparameters, which are set by trial and error in the traditional ANN model development process, and which greatly affect the network performance. In particular, the method using the AOMA-ANN (ONIP) model enables the development of models with lower error rates by removing some input parameters from the model and reduces the computational complexity of the model by removing unnecessary input parameter data from the model. The study focused on modeling Marsh funnel flow time and mini-slump results separately. Throughout the modeling study, a collinearity analysis was performed on a total of 13 input parameters, including cement fineness, 32, 45, and 60 micron sieve residue, zeta potential value, GA dosage, molecular weight, number of molecules per fineness, density, pH level, number of hydrogen bond acceptors, number of functional groups, and PCE dosage. For this purpose, variance inflation factors (VIF) were calculated. Input parameters with high VIF values were removed from the model one by one, and VIF values were calculated again and again. Parameters were reduced so that the VIF values of all parameters were less than 5. The VIF values of all experimental parameters for the Marsh funnel flow time and mini-slump models, the VIF values of all experimental parameters, and the Pearson correlation matrices for the input parameters used in the models are given in Figure 1 and Figure 2. 

**Figure 3:**
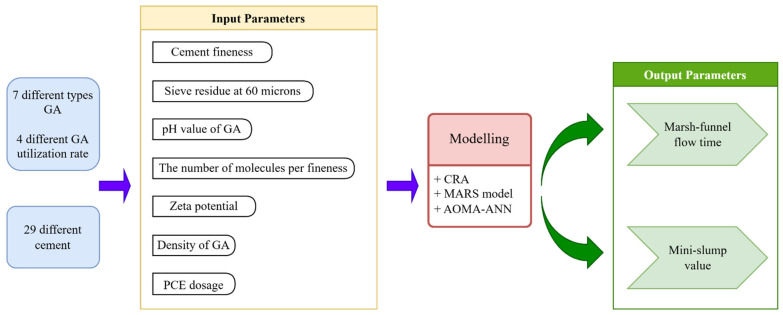
A schematic overview of the methodologies employed in the study.

To illustrate the methodology and data employed in the modeling process, refer to Figure 3 for a schematic summary. The data were normalized within a range of 0.1 to 0.9 and subsequently divided into training and testing datasets. For all analyses, various GAs, including TEA, TIPA, DEG, EG, M-TEA-1, and M-TEA-2 at different concentrations, were incorporated in the model testing phase. Specifically, 69% of the dataset was allocated for model creation and training, with the remaining 31% reserved for model testing.

In the context of this study, 197 data points were employed in modeling Marsh funnel flow time, while 189 data points were used for modeling mini-slump values. Consequently, 136 data points were designated for training the Marsh funnel flow-time model, leaving 61 data points for testing. Likewise, for modeling mini-slump values, 130 data points were used for training, and 59 data points for testing. Table 4 provides a detailed overview of the Marsh funnel flow time and mini-slump data used in this modeling study.

### 3.2. Classic Regression Analysis (CRA)

To establish the coefficients of equations that yielded the best alignment with the data collected from the experimental study, the initial step involved conducting a Classical Regression Analysis (CRA). In pursuit of this objective, six distinct regression functions, comprising linear, power, exponential, inverse, ln, and s functions, as presented in Equations (1)–(6), were employed.

(1) $$y_{linear}=w_{0}+w_{1}x_{1}+w_{2}x_{2}+w_{3}x_{3}+\cdots +w_{8}x_{7}$$

(2) $$y_{power}=w_{0}\cdot x_{1}^{w_{1}}\cdot x_{2}^{w_{2}}\cdot x_{3}^{w_{3}}\cdot \ldots \cdot x_{7}^{w_{7}}$$

(3) $$y_{exponantial}=w_{0}+\exp\left(w_{1}+w_{2}x_{1}+w_{3}x_{2}+w_{4}x_{3}+\cdots +w_{8}x_{7}\right)$$

(4) $$y_{inverse}=w_{0}+\frac{w_{1}}{x_{1}}+\frac{w_{2}}{x_{2}}+\frac{w_{3}}{x_{3}}+\cdots +\frac{w_{7}}{x_{7}}$$

(5) $$y_{Ln}=w_{0}+w_{1}\ln\left(x_{1}\right)+w_{2}\ln\left(x_{2}\right)+w_{3}\ln\left(x_{3}\right)+\cdots +w_{7}\ln\left(x_{7}\right)$$

(6) $$y_{s}=\exp\left(w_{0}+\frac{w_{1}}{x_{1}}+\frac{w_{2}}{x_{2}}+\frac{w_{3}}{x_{3}}+\cdots +\frac{w_{7}}{x_{7}}\right)$$

where (w_i_) is the unknown regression coefficient; (x_i_) and (y) are the independent and dependent variables, respectively.

In the initial phase of this study, a Classical Regression Analysis (CRA) was conducted to ascertain the coefficients of the regression equations outlined in Equations (1)–(6) for both Marsh funnel flow time and mini-slump data. Subsequently, the effectiveness of these methods was assessed by comparing the error values obtained from the training and testing datasets, using the established regression models along with those derived from the Multivariate Adaptive Regression Splines (MARS) and Artificial Neural Networks with Architecture Optimized through Metaheuristic Algorithms (AOMA-ANN) methods, as elaborated in the subsequent sections. 

### 3.3. Multivariate Adaptive Regression Splines (MARS)

MARS is a non-parametric regression method developed by Friedman [51]. Multivariate Adaptive Regression Splines (MARS) is a flexible and interpretable nonparametric regression technique that automatically captures non-linear relationships, interactions, and variable selection by constructing piecewise linear functions from input variables. MARS combines the advantages of traditional regression methods with the ability to handle complex data patterns and interactions efficiently. A MATLAB toolbox called ARESLab Version 1.13.0 [52] was used for the MARS model. 

### 3.4. Artificial Neural Networks with an Architecture Optimized by Metaheuristic Algorithms (AOMA-ANN) 

This section details the process of determining the optimal architecture for the Artificial Neural Network (ANN). In this study, the task of finding the best network architecture was transformed into an optimization challenge. The optimization problem’s objective function was defined as minimizing the average Mean Square Error (MSE) value across the training and test datasets of the model. This optimization process effectively converted the prior trial-and-error approach to identifying the optimal ANN network into a systematic optimization problem.

To train the feedforward network, the Levenberg–Marquardt algorithm, available in MATLAB toolboxes, was employed when invoking the objective function during the optimization procedure. Default settings were utilized for running this algorithm, with the only exception being the maximum training iteration value (epoch), which was capped at 30 to prevent excessive computational time during network optimization.

The performance assessment of the ANN network was based on two metrics: root mean square error (RMSE) and Nash–Sutcliffe efficiency coefficient (NS), as outlined in Equations (7) and (8).

(7) $$RMSE={\left[\frac{1}{n}\sum_{i=1}^{n}{\left(E_{i}-P_{i}\right)}^{2}\right]}^{1/2}$$

(8) $$NS=1-\frac{\sum_{i=1}^{n}{\left(E_{i}-P_{i}\right)}^{2}}{\sum_{i=1}^{n}{\left(E_{i}-\bar{E}\right)}^{2}}$$

In the context of this discussion, the variables are defined as follows: E_i_ represents either the experimental Marsh funnel flow time or the mini-slump value, $\bar{E}$ signifies the average of these values, P_i_ denotes the value derived from the model, and n denotes the total number of data observations. The Nash–Sutcliffe (NS) coefficient is a metric that ranges from −∞ to 1. An NS value of 1 indicates a model that fits perfectly, while NS values between 0.75 and 1 suggest a very good model fit. A range of 0.65 to 0.75 indicates a good model fit, while values between 0.50 and 0.65 suggest that the model is adequate. An NS value below 0.50 indicates that the model is inadequate.

In this study, two distinct optimization problems were formulated and designated AOMA-ANN(AIP) and AOMA-ANN(ONIP). In the AOMA-ANN(AIP) problem, the network was optimized using all input parameters, involving five design variables (NDV): the number of neurons in the first hidden layer, the number of neurons in the second hidden layer, the activation function in the first hidden layer, the activation function in the second hidden layer, and the activation function in the output layer. In essence, the AOMA-ANN(AIP) optimal design process determines the number of neurons in the hidden layer(s) of the network and selects the activation function types for these hidden layers and the output layer. The optimal activation functions were chosen from a pool of 12 different activation functions, including purelin, transit, logsig, elliotsig, hardlim, hardlims, satlin, satlins, poslin, tribas, radbasi, and radbasn.

**Figure 4:**
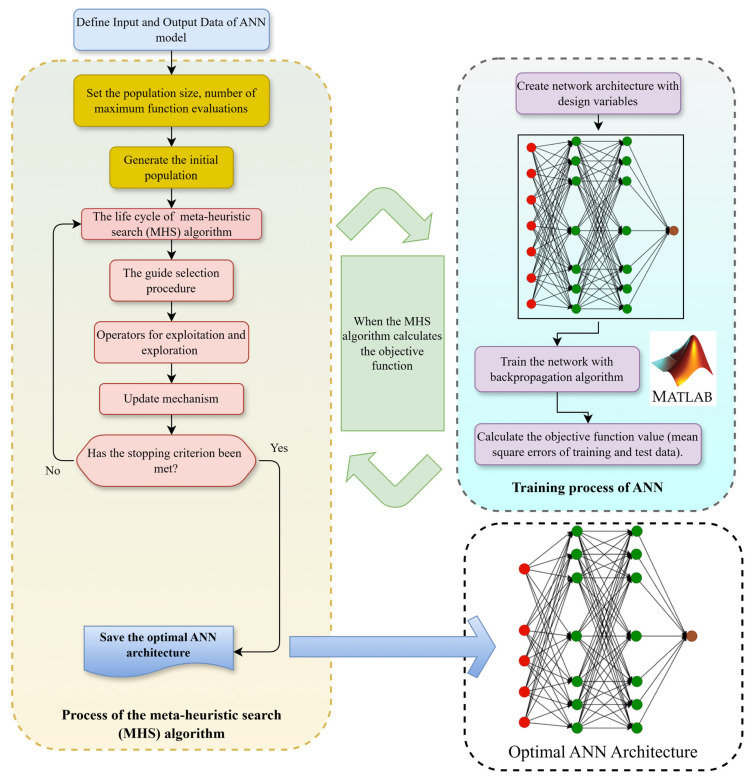
Optimization process of the Artificial Neural Network architecture.

In contrast, the AOMA-ANN(ONIP) model introduces the additional dimension of deciding which input parameters to incorporate to achieve the optimal network. In this model, binary design variables are introduced, which are equal in number to the input parameters. A design variable with a value of 1 indicates that the corresponding data will be used, while a value of 0 signifies that the data will not be utilized. In this study, a total of 12 (5 + 7) design variables (NDV) were employed in the AOMA-ANN(ONIP) model, corresponding to the seven input parameters. The lower and upper limits for these design variables, as defined in both the AOMA-ANN(AIP) and AOMA-ANN(ONIP) models, are detailed in Table 5. The optimization process for determining the Artificial Neural Network architecture is summarized in the flowchart presented in Figure 4. 

The objective of this optimization problem is to find a network that minimizes the errors in the training and test data. In the process, the network is trained by backpropagation algorithms, and the training process is performed with only training data. The optimization process monitors the errors in the training and test data and changes the design variables of the optimization process by taking these error values as a guide. The design parameters of the optimization problem are the number of neurons in the hidden layers, the activation functions in the hidden layers, and the output layer, as stated in Table 5 in the paper. In addition, in the AOMA-ANN (ONIP) model, switching variables are added to allow the input parameters to participate in the network. These variables are equal to the number of input parameters and allow the relevant input parameter to participate or not participate in the model. In this process, these design variables are updated by the metaheuristic algorithm-specific mechanisms of guide selection, exploitation, exploration, and update to create the network architecture with the lowest error in the solution space.

The simulation of the optimization problem involved the utilization of the Gaining–Sharing Knowledge (GSK)-based, JAYA, Symbiotic Organisms Search (SOS), Teaching–Learning-Based Artificial Bee Colony (TLABC), and Teaching–Learning-Based Optimization Algorithm (TLBO) algorithms, and their respective performances were subsequently compared. The parameter settings recommended by the developers of these metaheuristics were strictly adhered to and are summarized in Table 6. All algorithmic operations were executed on a workstation equipped with an Intel^®^ Xeon^®^ CPU E5-1650v3@3.50GHz processor. The termination criteria of the algorithm are based on the number of objective function evaluations (maxFEs) and the study was performed by setting maxFEs to 1000 times NDV, where NDV represents the number of design variables for network architecture optimization. For each sample, a total of 25 independent simulations were conducted. 

## 4. Assessment of Experimental Results

**Figure 5:**
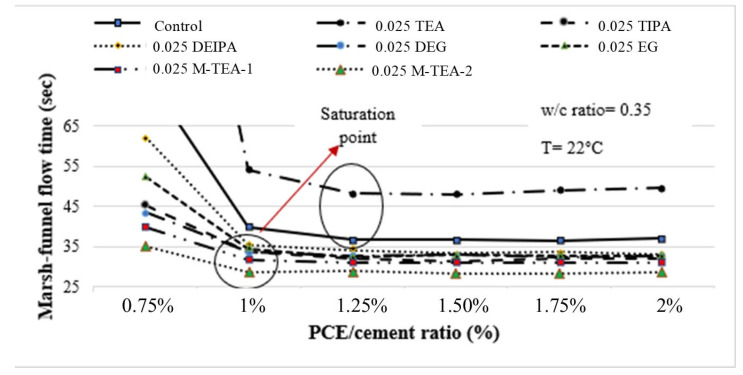
Marsh funnel flow times for both the control mixture and the mixtures containing 0.025% GA.

As can be seen in Table 3, the Marsh funnel flow time values of the mixtures decreased with the increase in the amount of PCE used, regardless of the PCE type and dosage. However, above a certain dosage, there was no significant change in the flow time values of the paste mixtures, despite the increase in the amount of PCE. This dosage represents the saturation point of PCE [4,11]. When the flow times at the saturation point are taken into consideration, increases in Marsh funnel flow time were observed with the increase in the GA usage rate in all other cementitious mixtures containing GA except for TEA. The flow performance was negatively affected due to the increase in narrow particles in the cement with the increase in the GA dosage. Similar findings were also obtained by Katsioti et al. [58]. Mardani-Aghabaglou et al. [16] reported that PCE adsorbed more fine cement particles, thus positively affecting flow performance. On the other hand, cements containing high rates of GA had a higher proportion of narrower particles at the same Blaine fineness. This may increase the PCE requirements [2,45]. However, it is thought that the presence of GA molecules on the surface of cement particles reduces the surface energy, causing a decrease in PCE adsorption. The more negative value of the zeta potential measured in the control cement compared to all cements with GA indicates that the intensity of PCE adsorption is lower in cements containing GA. In addition, as the GA utilization rate increases, the amount of PCE adsorption decreases as the surface energy of cement particles decreases at a higher rate. The decrease in flow performance with increasing GA utilization rate is associated with these conditions. For these reasons, the effect of GA on Marsh funnel flow performance was examined at the lowest usage rate of 0.25%. Then, the Marsh funnel flow time curves of paste mixtures containing 0.25% of different types of GA were compared with the control mixture (Figure 5).

Upon examining the flow time values at the saturation point, it becomes apparent that the Marsh funnel flow time for the mixture containing 0.025% TEA exceeded that of the control mixture by 35% (see Figure 5). This particular mixture exhibited the poorest flow properties, likely attributed to TEA’s role in accelerating hydration through interaction with C_3_A [59]. Since C_3_A and the ettringite phase are positively charged, the negatively charged PCE is primarily adsorbed to this phase. This reduces the dispersion effect and the flowability of the PCE [13,36]. Since the hydration of the C_3_A phase is accelerated in the presence of TEA, the amount of ettringite that is formed is higher [45,59,60]. This situation causes PCE attachment between the layers of ettringite or causes PCE to become embedded in the ettringite formed on the surface of C_3_A, resulting in a decrease in its effectiveness [13,43]. In contrast, the 0.025-M-TEA-2 mixture demonstrated superior performance, with a 28% reduction in flow time compared to the control mixture. This disparity can be attributed to GAs other than TEA, promoting smoother cement particle structures than the control cement and preventing agglomeration [1].

Sun et al. [7] noted that when higher dosages of GAs are used, the cement particle size distribution becomes finer, negatively impacting flow performance due to the reduced PCE adsorption in the presence of the GA. A similar behavior was observed in this study. As is evident from the results, while the positive effects of GA on fluidity were more pronounced at lower usage rates, their negative impact became increasingly dominant as usage rates escalated.

**Figure 6:**
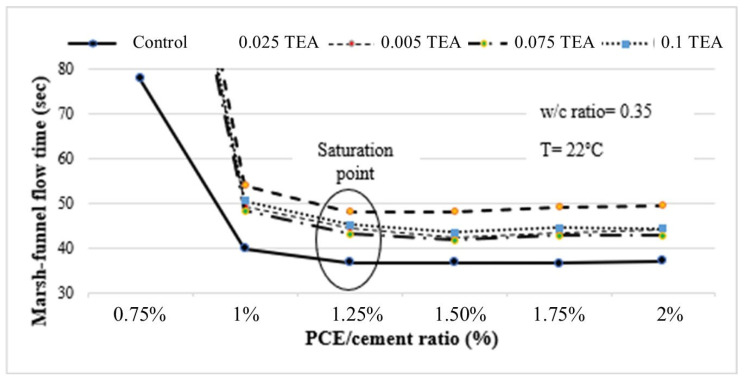
Marsh funnel flow-time curves for both the control mixture and the mixtures containing varying dosages of TEA.

Figure 6 presents Marsh funnel flow-time graphs for the control mixture without GA and paste mixtures containing various TEA dosages.

As illustrated in Figure 6, irrespective of the GA utilization rate, the Marsh funnel flow-time values for all TEA-containing mixtures were higher compared to those of the control mixture. Marsh funnel flow performance increases as the TEA utilization rate rises to 0.075 but experiences a decline beyond this threshold.

**Figure 7:**
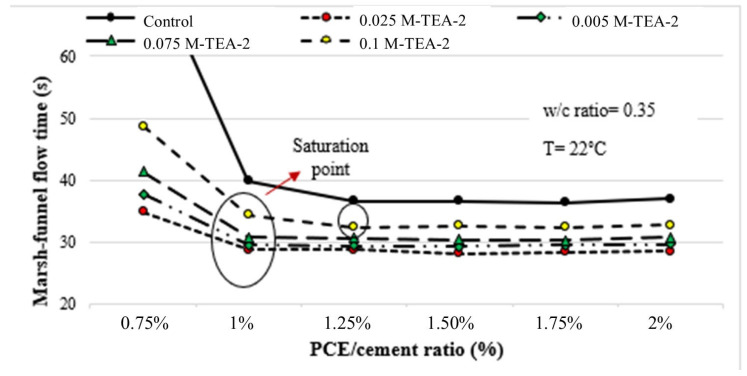
Marsh funnel flow-time curves for both the control mixture and the mixtures containing varying dosages of M-TEA-2.

A significant improvement in Marsh funnel flow-time performance was observed with the modification process applied to TEA. This enhancement is attributed to the introduction of stronger carboxyl groups, achieved through the modification process, which surpass the existing hydroxyl groups of TEA [1]. In this context, mixtures featuring M-TEA-2 exhibited a superior flow performance compared to those with M-TEA-1. The Marsh funnel flow-time curves for mixtures containing M-TEA-2 are presented in Figure 7.

As depicted in Figure 7, irrespective of the GA utilization rate, the flow performance of all mixtures containing M-TEA-2 exhibited superiority over the control mixture. The lowest Marsh funnel flow time value was observed at a 0.025% utilization rate, with an increase in flow time detected as the dosage increased. Despite a decrease in Zeta potential value with the rise in utilization rate, it is posited that the concurrent increase in Marsh-funnel flow time results from the augmented presence of fine particles, akin to the observations in other GAs. The use of GAs results in a reduction in zeta potential, primarily due to the adsorption of GA molecules onto cement particle surfaces. In the presence of GA, the adsorption capacity of PCE decreases, which can be attributed to both the refinement of particle size distribution and the pre-adsorption of GA on cement grains. This leads to an increase in Marsh funnel flow time.

## 5. Evaluation of Modeling Results

This section compares the findings obtained from the data modeling methods applied in this study. This section consists of four subsections. In the first subsection, the effect of Classical Regression Analysis (CRA) and MARS methods on Marsh funnel flow time and mini-slump value prediction models is analyzed. In the second subsection, the results obtained by running the ANN hyperparameter optimization procedure developed in this study using various heuristic search algorithms are compared, and the most successful heuristic algorithm is determined. In the third subsection, all the methods used are evaluated together, and the method with the highest prediction performance is presented. In the fourth and final subsection, the limitations of the obtained models are introduced.

### 5.1. Classical Regression Analysis and MARS Results

In this subsection, Marsh funnel flow-time prediction models and mini-slump value prediction models are constructed for the input parameters provided in Figure 3, with six different classical regression functions (linear, power, exponential, inverse, ln, and s) and the Multivariate Adaptive Regression Splines (MARS) method. The coefficients of the obtained classical regression functions and the basic functions and general equations of the MARS models are presented. In addition, the error values obtained from the Marsh Funnel flow time and mini-slump value prediction models were calculated for the experimental data for these models. Among the classical regression equations and the MARS method, the best models, with the lowest error values for Marsh funnel flow time and mini-slump value prediction, are determined.

The coefficients determined by the CRA method for modeling the Marsh funnel flow-time data are presented in Table 7. The functions and determined coefficients used to model the mini-slump data are summarized in Table 8. The basic functions and best-fit equations obtained with the MARS model for Marsh funnel flow time and mini-slump data are shown in Table 9 and Table 10, respectively.

Here, the variables $x_{1}$, $x_{2}$, $x_{3}$, $x_{4}$, $x_{5}$, $x_{6}$, and $x_{7}$ are symbolized as cement fineness (CF), 60-micron sieve residue, zeta potential value, number of molecules per fineness, density, pH, and PCE dosage, respectively.

The error values obtained when modeling Marsh funnel flow time and mini-slump data with regression equations and the MARS method are presented in Table 11 and Table 12, respectively. It was observed that the errors obtained using the MARS method for both Marsh funnel flow time and mini-slump were smaller compared to those obtained using the CRA method.

In the modeling of flow times, the NS value for the CRA function (EF), displaying the highest performance according to all data, was 0.859, whereas the NS value obtained with the MARS method increased to 0.882. The performance of the MARS method in mini-slump modeling significantly surpassed that of the CRA equations. In this model, the NS value for the CRA function (SF), with the highest performance of all data, was 0.722, while the NS value obtained with the MARS method increased to 0.878. 

### 5.2. ANN Modeling Results

In this section, in the first step, the ANN hyperparameter optimization introduced in “Section 3.4” is performed with various algorithms. The performance of the algorithms used to improve the predictive power of the analyzed models is analyzed. In the second stage, the networks that best represent the Marsh funnel flow time and mini-collapse prediction models are selected, and their network architectures are presented. These steps are introduced in more detail below, and the findings obtained from these steps are presented.

***Phase 1—Comparison of algorithm performances in ANN hyperparameter optimization:*** Hyperparameter optimization is designed in two different ways. These are as follows:

*Version 1—AOMA-ANN (AIP):* A model that takes into account all of the defined input parameters and optimizes the number of intermediate layers, the number of neurons in the intermediate layers, and the type of activation functions used in the layers from the ANN hypermeters.
*Version 2—AOMA-ANN (ONIP):* A model that, in addition to the capabilities of version 1, can reduce the input parameters used to reduce the prediction error.

As mentioned above, in the first version, called AOMA-ANN (AIP), only the network architecture and activation function type were optimized by using all input parameters. In the second version, called AOMA-ANN (ONIP), the selection of the input parameters to be used in the model is also included in the optimization process. In other words, the process also determines which input parameters should be used to minimize the error. Thus, input parameters that are unnecessary or degrade network performance are removed without additional statistical analysis. 

The optimization of AOMA-ANN (AIP) and AOMA-ANN (ONIP) models was performed with five different metaheuristic search algorithms, and their success in optimizing ANN hyperparameters was compared. The tested metaheuristic search algorithms were Gaining–Sharing Knowledge (GSK)-based, JAYA, Symbiotic Organisms Search (SOS), Teaching–Learning-Based Artificial Bee Colony (TLABC), and Teaching–Learning-Based Optimization Algorithm (TLBO). The maximum number of objective function evaluations was used as the stopping criterion of the algorithms, and this value was taken as 1000 times the number of design variables (maxFEs = 1000xD). For each algorithm, 21 independent simulations were performed. The number of design variables is D = 5 for AOMA-ANN (AIP) and D = 12 for AOMA-ANN (ONIP), as described in Section 3.4.

In the optimization process, the networks were trained using the training data with the backpropagation algorithm. In the optimization process, metaheuristic search algorithms monitor the errors in the training data of the trained network and the test data not used in the training of the network. In order to do this, the average of the mean squared errors of the training and test data of the network is used as the objective function of the optimization process. The performance of the algorithms is compared with a statistical study on the minimum error values obtained from independent simulations. The Friedman test [61], a non-parametric statistical test, was used to rank the success of the metaheuristic search algorithms in determining the optimal network structure. By applying the Friedman test to the error values obtained from independent simulations, the scores of the algorithms were obtained, and the metaheuristic algorithms were ranked according to their success. The Friedman test scores of the metaheuristic search algorithms are presented in Table 13.

From Table 13, it can be seen that the TLABC algorithm achieved the top ranking according to the Friedman score in the ANN architectural optimization (AIP) when using all input parameters for the Marsh funnel flow-time model. However, in the ANN architecture optimization with reduced input parameters (ONIP), the TLABC algorithm was also found to be the best-performing algorithm.

In the ANN architectural optimization (AIP) performed using all input parameters for the mini-slump model, the JAYA algorithm was the best algorithm according to the Friedman score. However, in ANN architecture optimization with reduced input parameters (ONIP), the TLBO algorithm emerges as the best-performing algorithm.

The fact that the output data characteristics of the Marsh funnel flow time and mini-slump models are different makes the search-space characteristics of the problems different from each other. Three different algorithms were successful in the optimization of these two models. According to the “no free lunch” theorem [62], there is no single algorithm that can successfully solve every optimization problem. These findings also support this theorem.

In summary, the TLABC algorithm is the most successful algorithm for the Marsh funnel flow time model, and the JAYA and TLBO algorithms are the most successful algorithms for the mini-slump model.

***Phase 2—Selection of the best ANN for the prediction models:*** In this stage, the networks with the smallest error for all the experimental data were selected from 21 independent simulations in the hyperparameter optimization performed using metaheuristic search algorithms. Among these networks, the algorithms that could determine the network with the best prediction performance were selected. Table 14 presents the MSE, RMSE, and NS values of the networks that obtained the smallest squared error value for all the experimental data in the independent simulations using the Marsh funnel flow time and mini-slump models.

According to Table 14, for the Marsh funnel flow-time model, the networks with the lowest error for all the experimental data were obtained with the TLABC algorithm using the AIP and ONIP methods. For the mini-slump ANN model, the networks with the lowest error for all the experimental data were obtained with the JAYA algorithm using the AIP and ONIP methods. When the values of the lowest errors obtained for all data in the Marsh funnel flow time and mini-slump models were analyzed, the NS value of the mini-slump model was shown to be smaller and the algorithms were revealed to have more difficulty reducing the error obtained using this model. This shows that it is more difficult to find the global minimum point in the search space when conducting a hyperparameter optimization of this model.

When the AIP and ONIP models were compared by examining the error values in Table 14, it could be observed that the errors in the models where the input parameters were optimized (ONIP) were smaller than those observed in the ANN architecture optimization (AIP) where all input parameters were used. Notably, the highest NS value obtained using the AIP method for the mini-slump model was 0.974, while it increased to 0.976 when using the ONIP method. This underscores the effectiveness of the developed input parameter reduction process.

**Figure 8:**
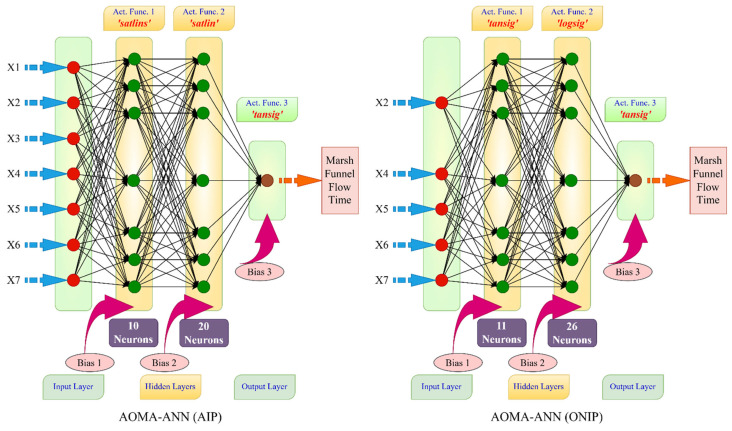
Optimum network architectures determined by the AIP and ONIP procedures for the Marsh funnel flow-time model.

**Figure 9:**
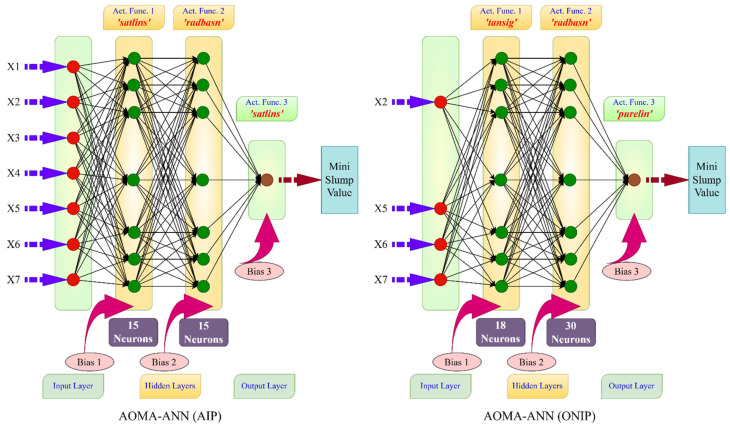
Optimum network architectures determined by the AIP and ONIP procedures for the mini-slump model.

In Table 14, the error values of the best networks with the lowest error rates (highest NS values) for all data are shown in bold. The network architectures that achieved these error rates are shown in Figure 8 and Figure 9 for the Marsh funnel flow time and mini-slump models, respectively.

Figure 8 and Figure 9 show that the algorithms in all models use two hidden layers as a result of optimization. However, in the Marsh funnel flow-time model, in the ANN architectural optimization (ONIP) that can reduce the input parameters, the input parameters that minimized the error were X2 (60-micron sieve residue), X4 (number of molecules pe fineness), X5 (density), X6 (pH), and X7 (PCE dosage). In the mini-slump model, in the ANN architecture optimization (ONIP) that can reduce the input parameters, the input parameters that minimize the error were X2 (60-micron sieve residue in cement), X5 (density), X6 (pH), and X7 (PCE dosage). Thus, it is understood that X2 (60-micron sieve residue in cement), X5 (density), X6 (pH), and X7 (PCE dosage) are the most effective input parameters and must be used in both models. 

### 5.3. Comparing Modeling Results

In this section, the errors obtained using the classical regression models (CRA), MARS model, and hyperparameter-optimized ANN models (AOMA-ANN AIP and ONIP) are compared. Thus, the model with the most efficient prediction performance of all the models tested is presented. 

In the comparison of all models employed in this study, RMSE values and NS values were determined according to the training, testing, and all data. The benchmark values of the CRA, MARS, and AOMA-ANN models regarding Marsh funnel flow time and mini-slump are presented in Table 15 and Table 16, respectively. The model results exhibiting the lowest error values (and the highest NS value) are highlighted in bold in the tables.

**Figure 10:**
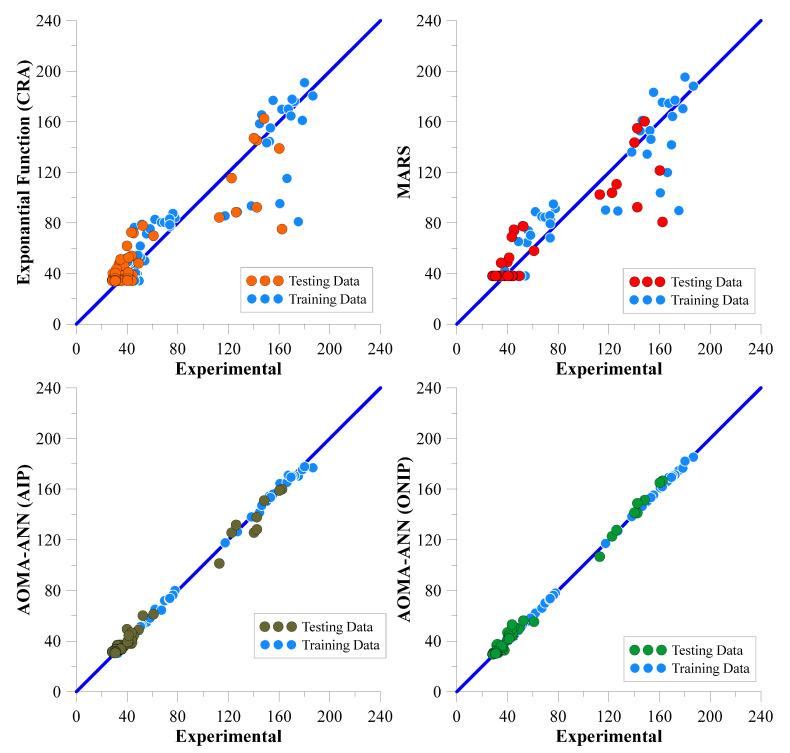
Scatter plots of predicted and experimental Marsh funnel flow times obtained from CRA-EF, MARS, AOMA-ANN(AIP), and AOMA-ANN(ONIP).

**Figure 11:**
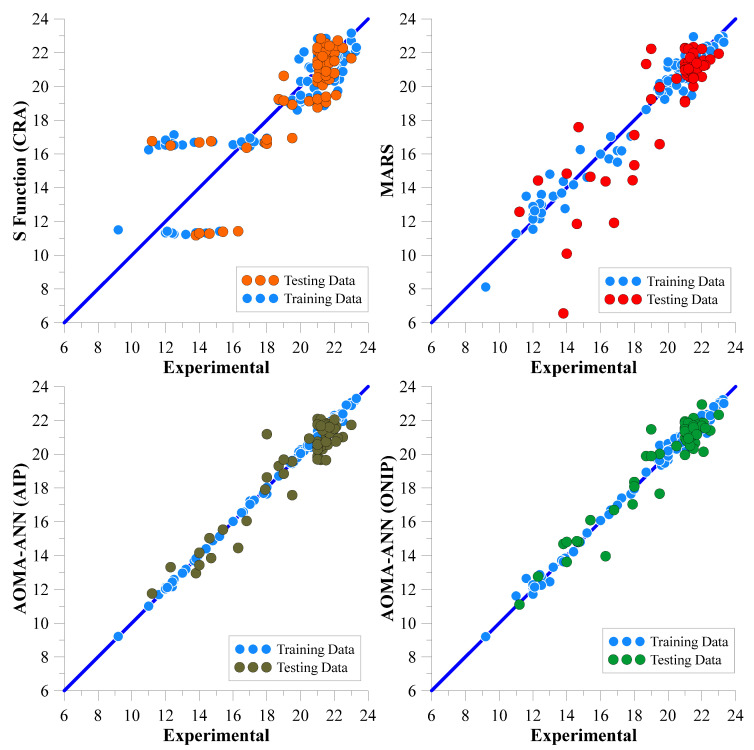
Scatter plots of predicted and experimental mini-slump values obtained from CRA-SF, MARS, AOMA-ANN(AIP), and AOMA-ANN(ONIP).

When analyzing the error and NS values, it is evident that the AOMA-ANN(ONIP) model demonstrates the best performance for both Marsh funnel flow time and mini-slump values. For Marsh funnel flow time, the LF, InvF, LnF, and SF models were identified as having low predictive power, while the PF, EF, MARS, and AOMA-ANN models exhibited high predictive power, as indicated by the NS value of the testing dataset. Considering the NS values of all data, the exponential function has the best prediction power among the classical regression functions. The scatter plots of the predictions obtained from the MARS and ANN models with exponential function (EF) are presented in Figure 10. In the case of mini-slump values, the CRA models (LF, PF, EF, InvF, LnF, and SF) were found to have low predictive power, while the MARS and AOMA-ANN models displayed high predictive power according to the NS value of the testing dataset. Considering the NS value of all data, the S function has the best prediction power among the classical regression functions. The scatter plots of the predictions obtained from the MARS and ANN models with S function (SF) are presented in Figure 11. 

When the error values given in Table 15 and Table 16 and the scatter diagrams shown in Figure 10 and Figure 11 are evaluated together, it can be seen that the Marsh funnel flow time and mini-slump values obtained for the AOMA-ANN (ONIP) model cluster much more around the zero-error line. Thus, this graphically shows that the AOMA-ANN (ONIP) model provides the strongest positive correlation between the predicted output values and the experimental output data.

### 5.4. Modeling Limitations 

In this subsection, the limitations of the study are defined by presenting the data ranges within which the models created in the study can work properly. The performance of the AOMA-ANN models employed in the study is notably high. However, it is essential to bear in mind that these models were developed based on a limited dataset. Therefore, the usage limits of the models are outlined in Table 17.

In summary, unlike most existing studies, which employ metaheuristics primarily for ANN training (leading to increased computational costs), this study focuses on optimizing ANN architecture using newer algorithms, such as GSK, JAYA, SOS, TLABC, and TLBO. This shift addresses the inefficiency of trial-and-error network design while reducing computational overhead compared to weight optimization via metaheuristics.

A critical gap identified in the literature is the lack of input parameter reduction techniques in cementitious system modeling. The ONIP model presented in this paper reduces input parameters in a way that minimizes model error and provides an optimization-based approach to improve model simplicity and accuracy. This is an innovation not found in previous studies. While prior studies predominantly rely on older algorithms (e.g., GA, PSO, and DE), this work evaluates newer and less-explored metaheuristics (e.g., GSK and TLBO), providing insights into their effectiveness in ANN architecture optimization for cement–PCE compatibility modeling. Most previous studies used only a single metaheuristic, making comparative performance evaluations difficult. This paper tested multiple algorithms and compares their performance.

While the proposed AOMA-ANN method shows promise, its applicability to other cementitious systems (e.g., those with different admixtures or curing conditions) remains to be validated, as most prior studies focus on specific scenarios. Despite avoiding metaheuristic-based training, the computational cost of architecture optimization (especially with multiple algorithms) could be quantitatively compared with that of existing hybrid ANN approaches.

By contextualizing the findings within the limitations and advancements of prior studies, the discussion can more effectively highlight this work’s contributions: (1) a novel metaheuristic application for Artificial Neural Network (ANN) architecture optimization is developed, (2) the input parameters required for streamlined modeling are reduced, and (3) untested algorithms are introduced in cementitious system modeling. However, further comparative studies and broader validation are necessary to solidify these advancements.

## 6. Conclusions

In this study, the compatibility of PCE with cements produced using various types and dosages of GA was investigated through experimental and statistical methods. The research employed Classical Regression Analysis (CRA), Multivariate Adaptive Regression Splines (MARS), and Artificial Neural Networks with Architecture Optimized by Metaheuristic Algorithms (AOMA-ANN). Additionally, two innovative approaches, AOMA-ANN (AIP) and AOMA-ANN (ONIP), were developed to identify the optimal Artificial Neural Network architecture. 

The study’s findings, based on the utilized materials and applied methods, can be summarized as follows:

- Except for TEA, the flow performance of the mixtures was adversely affected by the increase in GA usage. This negative impact was attributed to a reduction in PCE adsorption and an increase in fine particles. Mixtures containing TEA exhibited the lowest flow performance.
- The AOMA-ANN (ONIP) model demonstrated superior performance, evidenced by their achieving the lowest error values (highest NS values) for both Marsh funnel flow time and mini-slump. The AOMA-ANN(ONIP) process systematically determined the input parameters required for error minimization, removing some input parameters that could compromise network performance without additional statistical analysis.
- Among the metaheuristic search algorithms used in the AOMA-ANN (AIP) model, the most effective algorithms in terms of hyperparameter optimization are the TLABC and JAYA algorithms, while the most effective algorithms in the AOMA-ANN (ONIP) model are the TLABC and TLBO algorithms.
- The models developed with the MARS method were significantly better in terms of their Marsh funnel flow time and mini-slump values than those derived from CRA equations. The error values of the MARS models were notably lower than those of CRA equations.
- The Marsh funnel flow time and mini-slump models created with AOMA-ANN(ONIP) exhibited the lowest scatter and accordingly were shown to have the best performance. Their predictive power surpassed that of other models.
- The AOMA-ANN model, utilized for the first time in the statistical modeling of the experimental data in this study, emerged as the most suitable data-modeling method. Predictions from this model are expected to yield comparable results, reducing the application time, materials, and labor required for extensive Marsh funnel experiments.

The validation of the performance of the AOMA-ANN (ONIP) model developed in this study, especially by applying it to data obtained from real-world applications, will be the subject of our future work.

## Figures and Tables

**Table 2:** Particle size distribution and Zeta potential values of the cements.

Types of Cement	Zeta Potential Values	Blaine Fineness (cm^2^/g)	Residue on 32 µm Sieve (%)	Residue on 45 µm Sieve (%)	Residue on 60 µm Sieve (%)
Control	−12.0	4100	31.9	10.4	1.2
0.025 TEA	−8.63	4110	26.5	7.6	0.8
0.05 TEA	−5.41	4115	18.0	4.7	0.7
0.075 TEA	−8.02	4100	16.6	4.0	0.5
0.1 TEA	−9.1	4090	14.0	3.1	0.3
0.025 TIPA	−6.24	4100	24.9	8.2	0.9
0.05 TIPA	−4.8	4180	19.0	4.9	0.8
0.075 TIPA	−3.0	4200	11.7	2.9	0.5
0.1 TIPA	−2.05	4140	9.6	2.1	0.2
0.025 DEIPA	−9.23	4080	25.8	9.5	0.8
0.05 DEIPA	−7.12	4090	21.0	5.8	0.7
0.075 DEIPA	−4.56	4100	15.2	3.2	0.5
0.1 DEIPA	−3.59	4020	13.2	3.0	0.4
0.025 DEG	−8.55	4110	23.8	8.2	0.9
0.05 DEG	−7.09	4140	16.7	6.0	0.7
0.075 DEG	−3.82	4190	12.5	4.8	0.6
0.1 DEG	−4.64	4150	9.5	3.1	0.4
0.025 EG	−9.41	4160	25.4	9.3	0.9
0.05 EG	−7.84	4190	25.8	8.1	0.9
0.075 EG	−5.12	4180	22.3	6.2	0.9
0.1 EG	−4.89	4160	17.3	4.3	0.7
0.025 M-TEA-1	−5.74	4120	24.6	9.0	0.9
0.05 M-TEA-1	−8.35	4070	21.0	6.9	0.6
0.075 M-TEA-1	−8.45	4100	14.8	3.5	0.4
0.1 M-TEA-1	−7.88	4050	14.5	3.4	0.3
0.025 M-TEA-2	−2.79	4080	28.2	11.2	1.1
0.05 M-TEA-2	−1.76	4080	26.2	10.2	1.0
0.075 M-TEA-2	−1.1	4060	24.4	9.0	0.9
0.1 M-TEA-2	−0.897	4050	23.5	9.0	0.8

**Table 3:** Some chemical properties of the GAs and PCE used.

Type of Admixture	Alkaline Content (%) (Na_2_O)	Density (g/cm^3^)	Solid Content (%)	Chloride Content (%)	pH. 25 °C	Number of Functional Groups
TEA	<10	1.095	50.0	<0.1	10.5	3
TIPA	<10	1.124	50.0	<0.1	10.8	3
DEIPA	<10	1.079	50.0	<0.1	9.7	3
DEG	<10	1.118	50.0	<0.1	7.2	2
EG	<10	1.260	50.0	<0.1	8.2	2
M-TEA1	<10	1.206	50.0	<0.1	5.6	2
M-TEA2	<10	1.166	50.0	<0.1	2.6	2
PCE	<10	1.097	36.0	<0.1	3.8	-

**Table 4:** Marsh funnel flow time and mini-slump values of paste mixtures.

		PCE/Cement Ratio						
Mixtures		0.50%	0.75%	1%	1.25%	1.50%	1.75%	2%
**C**	**Flow time (s)**	186.7	77.79	39.82	36.66 **	36.62	36.41	36.98
	**Mini-slump (cm)**	9.2	12.5	22.2	**21.5**	23.25	23	23
**0.025 TEA**	**Flow time (s)**	- *	175.24	54	**48.09**	48.06	49.06	49.53
	**Mini-slump (cm)**	-	11.6	19.5	**20**	20.5	21	22
**0.05 TEA**	**Flow time (s)**		162.36	49.12	**44.25**	42.41	43.37	43.91
	**Mini-slump (cm)**	-	12.3	19.5	**21**	21.5	21.5	21.5
**0.075 TEA**	**Flow time (s)**	-	160.61	48.22	**43.13**	41.75	42.72	42.68
	**Mini-slump (cm)**	-	12.4	20	**22.3**	22.4	22.7	23.3
**0.1 TEA**	**Flow time (s)**	-	166.25	50.45	**45.22**	43.47	44.53	44.21
	**Mini-slump (cm)**	-	12	20	**22**	22.5	21.7	22.3
**0.025 TIPA**	**Flow time (s)**	142.4	45.12	**33.72**	31.88	31.43	32.13	32.03
	**Mini-slump (cm)**	13.8	16.8	**21**	21.3	21.1	21.6	21.2
**0.05 TIPA**	**Flow time (s)**	152.4	56.5	**37.66**	36.62	35.96	37.01	36.67
	**Mini-slump (cm)**	13.9	17	**21.4**	21.4	20.6	19.9	20.2
**0.075 TIPA**	**Flow time (s)**	178.4	74.07	**42.03**	41.41	40.03	40.28	40.78
	**Mini-slump (cm)**	13.2	16.6	**19.5**	20.6	20.6	21	21.2
**0.1 TIPA**	**Flow time (s)**	180.1	76.22	**42.56**	39.76	40.46	40.28	40.95
	**Mini-slump (cm)**	12.5	16	**20.9**	21.6	21	21	21.2
**0.025 DEIPA**	**Flow time (s)**	155.3	62	**35.53**	34.13	33.34	33.62	33.12
	**Mini-slump (cm)**	**-**	13	**21**	22	22.1	22.2	22
**0.05 DEIPA**	**Flow time (s)**	162.2	67.21	36.21	**33.95**	34.12	34.45	34.23
	**Mini-slump (cm)**	-	12.5	19.4	**21.3**	22	22.1	22.1
**0.075 DEIPA**	**Flow time (s)**	167.3	69.61	38.1	**34.51**	34.14	34.57	34.85
	**Mini-slump (cm)**	-	12	19.6	**20.4**	21.1	22	21.8
**0.1 DEIPA**	**Flow time (s)**	172.2	73.4	37.41	**34.83**	34.19	34.6	35.31
	**Mini-slump (cm)**	-	11	19.8	**20.8**	21.4	21.9	22
**0.025 DEG**	**Flow time (s)**	140.2	43.41	**33.6**	32.62	32.97	32.45	32.75
	**Mini-slump (cm)**	14	17.9	**20.5**	21.5	21.5	21.2	21.6
**0.05 DEG**	**Flow time (s)**	144.6	45.75	**35.97**	33.72	34.02	33.92	34.09
	**Mini-slump (cm)**	14.4	18	**21.5**	21.8	22	21.9	22
**0.075 DEG**	**Flow time (s)**	150.2	55.37	**38.93**	36.41	36.62	36.34	36.73
	**Mini-slump (cm)**	12.4	17.8	**20.8**	22	21.8	21.8	21.9
**0.1 DEG**	**Flow time (s)**	170.3	73.65	42.49	**39.12**	38.41	38.82	38.95
	**Mini-slump (cm)**	12	16.5	19.5	**21**	22	21.8	21.9
**0.025 EG**	**Flow time (s)**	148.2	52.47	**34.53**	32.28	32.88	32.62	32.73
	**Mini-slump (cm)**	**-**	14.7	**18.7**	19	21	21	21.2
**0.05 EG**	**Flow time (s)**	153.2	58.19	**35.85**	34.75	34.56	34.2	34.8
	**Mini-slump (cm)**	-	14.8	**20**	21.5	21.5	21.7	22
**0.075 EG**	**Flow time (s)**	160.2	60.91	**37.57**	35.86	35.5	35.85	35.63
	**Mini-slump (cm)**	-	14	**19**	21	21.7	22	22.1
**0.1 EG**	**Flow time (s)**	169.4	73.71	**40.53**	39.18	38.84	39.01	38.72
	**Mini-slump (cm)**		13.7	**18.7**	21	21.2	21.7	21.4
**0.025 M-TEA-1**	**Flow time (s)**	122.5	39.95	**31.59**	31.06	30.87	30.99	31.02
	**Mini-slump (cm)**	14.6	18	**21.5**	21.7	21	21.2	21
**0.05 M-TEA-1**	**Flow time (s)**	-	51.68	**36**	35.06	34.06	34.64	34.35
	**Mini-slump (cm)**	-	17.25	**21.3**	21.5	22	21.7	21.7
**0.075 M-TEA-1**	**Flow time (s)**	-	127.11	50.61	**46.76**	45.31	45.81	46.02
	**Mini-slump (cm)**	-	12	20.5	**21.7**	21.7	21	21.2
**0.1 M-TEA-1**	**Flow time (s)**	**-**	142.45	43.56	**40.94**	40.53	40.06	40.41
	**Mini-slump (cm)**	-	11.2	21	**21.5**	22	21.5	21.5
**0.025 M-TEA-2**	**Flow time (s)**	112.8	34.9	**28.75**	28.81	28.19	28.37	28.53
	**Mini-slump (cm)**	15.4	18	**21.5**	22	23	22.5	22.2
**0.05 M-TEA-2**	**Flow time (s)**	117.4	37.8	**29.56**	29.44	29.21	29.56	29.63
	**Mini-slump (cm)**	15.2	18	**20**	22.5	21	21.3	21
**0.075 M-TEA-2**	**Flow time (s)**	126.1	41.3	**30.81**	30.63	30.43	30.21	30.72
	**Mini-slump (cm)**	16.3	19.5	**22.1**	21.5	21.3	21.7	21.2
**0.1 M-TEA-2**	**Flow time (s)**	138.1	48.7	**34.41**	32.35	32.63	32.42	32.78
	**Mini-slump (cm)**	12.1	17	**21**	21.5	21.3	21.7	21.5

* Measurement could not be taken. ** Saturation point flow times are highlighted in bold.

**Table 5:** Lower and upper limits of the design variables and increment values, and the range of possible values.

Design Variable	Lower Limit	Upper Limit	Increment	Number of Possible Values
D_1_: Number of neurons in the first hidden layer	2	30	1	29
D_2_: Number of neurons in the second hidden layer	0	30	1	31
D_3_: Activation function in the first hidden layer	{purelin, tansig, logsig, elliotsig, hardlim, hardlims, satlin, satlins, poslin, tribas, radbasi, radbasn}			12
D_4_: Activation function in the second hidden layer				12
D_5_: Output layer activation function				12
D_6_ *: 1. Decision variable for utilization of input data	0	1	1	2
…	…	…	…	…
D_(6+NDV)_ * NDV. Decision variable for utilization of input data	0	1	1	2

* For only AOMA-ANN(ONIP) model. NDV is the number of design variables for ANN architecture optimization.

**Table 6:** Settings for algorithm parameters.

Algorithm	Reference	Settings
GSK	[53]	Population size = 100, p = 0.1, $k_{f}$ = 0.5, $k_{r}$ = 0.9, K = 10
JAYA	[54]	Population size = 50
SOS	[55]	Ecosystem size = 50
TLABC	[56]	Number of food sources (NP) = 50, limit = 200, Scale factor (F) = rand
TLBO	[57]	Population size = 50

**Table 7:** Coefficients obtained from CRA for Marsh funnel flow time.

		Models					
		Linear Function (LF)	Power Function (PF)	Exponential Function (EF)	Inverse Function (InvF)	Ln Function (LnF)	S Function (SF)
Coefficients	w0	0.672686	0.067125	0.129688	−0.01604	−0.03504	−2.23811
	w1	0.000127	−0.00522	1.578782	−0.00051	0.004193	0.00348
	w2	−0.10709	−0.20029	−0.01263	0.009211	−0.04366	0.032356
	w3	−0.07709	−0.32749	−1.07705	0.035599	−0.05802	0.138471
	w4	0.026625	0.08254	−1.05479	−0.0051	0.006858	−0.03734
	w5	−0.10448	−0.07219	0.312201	−0.00568	−0.0143	0.011679
	w6	0.059809	0.173447	−0.73144	−0.02005	0.034241	−0.0938
	w7	−0.57552	−0.9488	0.477011	0.073521	−0.2709	0.186458
	w8			−8.06557			

**Table 8:** Coefficients obtained from CRA for mini-slump values.

		Models					
		Linear Function (LF)	Power Function (PF)	Exponential Function (EF)	Inverse Function (InvF)	Ln Function (LnF)	S Function (SF)
Coefficients	w0	0.3653394	0.905302	−16.577439	0.886149	0.8901377	−0.0077721
	w1	0.0324581	0.0124977	2.833863	−0.0064834	0.0235283	−0.004288
	w2	−0.0002337	−0.0048646	0.0020997	0.0011546	−0.0061773	0.0010272
	w3	−0.0294216	−0.0256414	−0.0026935	−0.0049123	−0.0016362	0.0096925
	w4	−0.0275952	0.0032983	−0.0021603	0.0009593	−0.0120337	−0.0048578
	w5	0.0844444	0.0454036	−0.0022321	−0.0191991	0.0490472	−0.0249233
	w6	−0.0653068	−0.0519783	0.0034666	0.0255447	−0.0522755	0.0271541
	w7	0.5895366	0.3988167	−0.005128	−0.0692647	0.2687517	−0.1521553
	w8			0.0337381			

**Table 9:** Basic functions and equations obtained from MARS for Marsh funnel flow time.

Basic Functions
BF1 = max(0, 1 − x7) BF2 = BF1 * max(0, x2 − 0.9) BF3 = BF1 * max(0, 0.9 − x2) BF4 = BF1 * max(0, x3 + 2.05) BF5 = BF1 * max(0, −2.05 − x3) BF6 = max(0, 0.75 − x7) BF7 = BF1 * max(0, 10.8 − x6)
Marsh-funnel flow time = 38.149 + 70.296 * BF1 + 518.51 * BF2 + 224.02 * BF3 + 140.45 * BF4 + 18.102 * BF5 + 174.64 * BF6 − 17.882 * BF7

**Table 10:** Basic functions and equations obtained from the MARS model for mini-slump values.

Basic Functions
BF1 = C(x6|+1, 4.915, 7.2, 8.85) BF2 = C(x6|−1, 4.915, 7.2, 8.85) BF3 = C(x7|+1, 0.625, 0.75, 0.875) * C(x6|+1, 1.315, 2.63, 4.915) BF4 = C(x7|−1, 0.875, 1, 1.125) * C(x1|+1, 4050, 4080, 4085) BF5 = C(x7|−1, 0.875, 1, 1.125) * C(x1|−1, 4050, 4080, 4085) BF6 = C(x7|−1, 0.875, 1, 1.125) * C(x1|+1, 4085, 4090, 4115) BF7 = C(x7|−1, 0.875, 1, 1.125) * C(x1|−1, 4115, 4140, 4160) BF8 = C(x7|+1, 0.875, 1, 1.125) * C(x6|+1, 8.85, 10.5, 10.65) BF9 = C(x4|+1, 0.47076, 0.94152, 3.386) * C(x7|−1, 1.125, 1.25, 1.625) BF10 = C(x7|−1, 0.625, 0.75, 0.875) * C(x1|−1, 4160, 4180, 4190) BF11 = C(x4|−1, 0.47076, 0.94152, 3.386) * C(x1|+1, 4050, 4080, 4085) BF12 = C(x4|−1, 0.47076, 0.94152, 3.386) * C(x1|−1, 4050, 4080, 4085) BF13 = C(x7|+1, 0.875, 1, 1.125) * C(x5|+1, 0.555, 1.11, 1.115) BF14 = C(x7|+1, 1.125, 1.25, 1.625) BF15 = C(x4|+1, 0.47076, 0.94152, 3.386) * C(x5|+1, 1.14, 1.16, 1.18) BF16 = C(x7|+1, 0.875, 1, 1.125) * C(x3|+1, −8.56, −8.02, −4.4585) BF17 = C(x7|+1, 0.875, 1, 1.125) * C(x3|-1, −8.56, −8.02, −4.4585) BF18 = C(x7|+1, 0.625, 0.75, 0.875) * C(x3|+1, -10.55, −9.1, −8.56) BF19 = C(x7|+1, 0.625, 0.75, 0.875) * C(x3|−1, −10.55, −9.1, −8.56) BF20 = C(x5|+1, 1.115, 1.12, 1.14) BF21 = C(x5|−1, 1.115, 1.12, 1.14)
Mini-slump = 21.144 − 0.19824 * BF1 + 0.43059 * BF2 + 0.37666 * BF3 − 1.5606 * BF4 +0.16825 * BF5 + 1.5794 * BF6 − 0.40074 * BF7 − 1.9121 * BF8 − 1.5543 * BF9 + 0.17399 * BF10 − 0.0511 * BF11 − 0.047408 * BF12 + 7.4156 * BF13 − 1.3511 * BF14 − 63.353 * BF15 + 0.52832 * BF16 − 1.0831 * BF17 − 0.51918 * BF18 + 1.878 * BF19 − 0.11206 * BF20 − 1.7269 * BF21

**Table 11:** RMSE, MAE, and NS values of CRA and MARS in training, testing, and all data for Marsh funnel flow times.

Model	Training Data		Testing Data		All Data	
	RMSE	NS	RMSE	NS	RMSE	NS
LF	30.82	0.490	28.72	0.423	30.19	0.477
PF	15.89	0.864	17.82	0.778	16.52	0.843
EF	14.86	0.882	17.29	0.791	15.65	0.859
InvF	16.85	0.848	19.93	0.722	17.86	0.817
LnF	22.60	0.726	23.24	0.622	22.80	0.701
SF	17.20	0.841	19.14	0.744	17.83	0.817
MARS	13.46	0.903	16.19	0.817	14.36	0.882

**Table 12:** RMSE, MAE, and NS values of CRA and MARS in training and testing sets for mini-slump values.

Model	Training Data		Testing Data		All Data	
	RMSE	NS	RMSE	NS	RMSE	NS
LF	2.14	0.598	1.96	0.513	2.09	0.578
PF	1.86	0.696	1.77	0.601	1.84	0.674
EF	2.16	0.594	1.98	0.502	2.10	0.573
InvF	1.87	0.694	1.80	0.588	1.85	0.669
LnF	1.71	0.744	1.69	0.639	1.70	0.719
SF	1.65	0.763	1.79	0.592	1.70	0.722
MARS	0.60	0.968	1.80	0.587	1.13	0.878

**Table 13:** Friedman test scores for the metaheuristic search algorithms.

	Model Type	Algorithms Friedman Scores				
		GSK	JAYA	SOS	TLABC	TLBO
Marsh Funnel Flow-Time ANN Model	AOMA-ANN (AIP)	2.95	3.38	3.24	2.33	3.10
	AOMA-ANN (ONIP)	4.05	3.38	3.10	2.14	2.33
Mini-Slump ANN Model	AOMA-ANN (AIP)	2.43	2.14	3.10	3.48	3.86
	AOMA-ANN (ONIP)	3.33	2.57	3.90	3.02	2.17

**Table 14:** MSE, RMSE, and NS values of the best networks using the Marsh funnel flow time and mini-slump ANN models.

Model	Method	Algorithm	Training Dataset			Testing Dataset			All Datasets		
			MSE	RMSE	NS	MSE	RMSE	NS	MSE	RMSE	NS
Marsh-Funnel Flow Time ANN Model	AOMA-ANN (AIP)	GSK	1.28	1.13	0.999	24.80	4.98	0.983	8.56	2.93	0.995
		JAYA	0.92	0.96	1.000	27.34	5.23	0.981	9.10	3.02	0.995
		SOS	2.18	1.47	0.999	20.85	4.57	0.985	7.96	2.82	0.995
		**TLABC**	2.20	1.48	0.999	17.11	4.14	0.988	**6.81**	**2.61**	**0.996**
		TLBO	0.69	0.83	1.000	27.17	5.21	0.981	8.89	2.98	0.995
	AOMA-ANN (ONIP)	GSK	0.35	0.59	1.000	15.15	3.89	0.989	4.94	2.22	0.997
		JAYA	0.63	0.80	1.000	13.17	3.63	0.991	4.51	2.12	0.997
		SOS	0.49	0.70	1.000	17.00	4.12	0.988	5.60	2.37	0.997
		**TLABC**	0.41	0.64	1.000	9.44	3.07	0.993	**3.21**	**1.79**	**0.998**
		TLBO	0.87	0.93	1.000	11.06	3.32	0.992	4.03	2.01	0.998
Mini-Slump ANN Model	AOMA-ANN (AIP)	GSK	0.03	0.18	0.997	0.83	0.91	0.894	0.28	0.53	0.973
		**JAYA**	0.02	0.14	0.998	0.83	0.91	0.895	**0.27**	**0.52**	**0.974**
		SOS	0.06	0.25	0.995	0.75	0.86	0.905	0.28	0.53	0.973
		TLABC	0.10	0.31	0.992	0.82	0.91	0.896	0.32	0.57	0.969
		TLBO	0.04	0.20	0.997	0.89	0.94	0.887	0.31	0.55	0.971
	AOMA-ANN (ONIP)	GSK	0.06	0.23	0.995	0.69	0.83	0.913	0.25	0.50	0.976
		**JAYA**	0.08	0.28	0.993	0.61	0.78	0.923	**0.24**	**0.49**	**0.976**
		SOS	0.05	0.22	0.996	0.72	0.85	0.909	0.26	0.51	0.975
		TLABC	0.09	0.30	0.992	0.65	0.81	0.918	0.27	0.52	0.974
		TLBO	0.02	0.15	0.998	0.78	0.88	0.902	0.26	0.51	0.975

**Table 15:** RMSE and NS values of Marsh funnel flow time obtained by the CRA, MARS, and AOMA-ANN models.

		Models								
Data	Error	Classical Regression Analysis						MARS	AOMA-ANN	
		LF	PF	EF	InvF	LnF	SF		AIP	ONIP
Training	RMSE	30.82	15.89	14.86	16.85	22.60	17.20	13.46	2.20	0.41
	NS	0.490	0.864	0.882	0.848	0.726	0.841	0.903	0.999	1.000
Testing	RMSE	28.72	17.82	17.29	19.93	23.24	19.14	16.19	17.11	9.44
	NS	0.423	0.778	0.791	0.722	0.622	0.744	0.817	0.988	0.993
All	RMSE	28.72	17.82	17.29	19.93	23.24	19.14	16.19	6.81	**3.21**
	NS	0.423	0.778	0.791	0.722	0.622	0.744	0.817	0.996	**0.998**

**Table 16:** RMSE and NS mini-slump values obtained by the CRA, MARS, and AOMA-ANN models.

		Models								
Data	Error	Classical Regression Analysis						MARS	AOMA-ANN	
		LF	PF	EF	InvF	LnF	SF		AIP	ONIP
Training	RMSE	2.14	1.86	2.16	1.87	1.71	1.65	0.60	0.02	0.08
	NS	0.598	0.696	0.594	0.694	0.744	0.763	0.968	0.998	0.993
Testing	RMSE	1.96	1.77	1.98	1.80	1.69	1.79	1.80	0.83	0.61
	NS	0.513	0.601	0.502	0.588	0.639	0.592	0.587	0.895	0.923
All	RMSE	2.09	1.84	2.10	1.85	1.70	1.70	1.13	0.27	**0.24**
	NS	0.578	0.674	0.573	0.669	0.719	0.722	0.878	0.974	**0.976**

**Table 17:** Limits of models’ use.

Input Parameters							
Limit	X_1_	X_2_	X_3_	X_4_	X_5_	X_6_	X_7_
Minimum	4020	0.2	−12	0	0	0	0.5
Maximum	4200	1.2	−0.897	5.830	1.2	10.8	2
X_1_: Cement fineness X_2_: Sieve residue at 60 microns X_3_: Zeta potential X_4_: Number of molecules per fineness					X_5_: Density of GA X_6_: pH value of GA X_7_: PCE dosage (%)		

## Data Availability

The data presented in this study are available on request from the corresponding author due to the data used in this study is confidential.

## Nomenclature

AI	Artificial Intelligence
ANN	Artificial Neural Network
CNN	Convolutional Neural Network
CO_2_	Carbon Dioxide
CRA	Classical Regression Analysis
DEG	Diethylene Glycol
DEIPA	Diethanol isopropanol amine
EG	Ethylene glycol
LF	Linear Regression Function
MAE	Mean Absolute Error
MARS	Multivariate Adaptive Regression Splines
MARCH	Multiple Additive Regression Trees
M-TEA	Modified Triethanolamine
GA	Grinding Aid
PCE	Polycarboxylate Ether-Based Water-Reducing Admixture
PF	Power Regression Function
RF	Random Forest
RMSE	Root Mean Square Error
TEA	Triethanolamine
TIPA	Triisopropanolamine
ANN	Artificial Neural Networks
